# Use of Non-Steroidal Anti-Inflammatory Drugs and Prostate Cancer Risk: A Population-Based Nested Case-Control Study

**DOI:** 10.1371/journal.pone.0016412

**Published:** 2011-01-28

**Authors:** Salaheddin M. Mahmud, Eduardo L. Franco, Donna Turner, Robert W. Platt, Patricia Beck, David Skarsgard, Jon Tonita, Colin Sharpe, Armen G. Aprikian

**Affiliations:** 1 Department of Oncology, McGill University, Montreal, Canada; 2 Department of Surgery (Urology), McGill University, Montreal, Canada; 3 Department of Community Health Sciences, University of Manitoba, Winnipeg, Canada; 4 Department of Epidemiology, Biostatistics, and Occupational Health, McGill University, Montreal, Canada; 5 Population Health Branch, Saskatchewan Ministry of Health, Regina, Canada; 6 Division of Radiation Oncology, University of Calgary and Tom Baker Cancer Centre, Calgary, Canada; 7 Population Health Division, Saskatchewan Cancer Agency, Regina, Canada; Health Canada, Canada

## Abstract

**Background:**

Despite strong laboratory evidence that non-steroidal anti-inflammatory drugs (NSAIDs) could prevent prostate cancer, epidemiological studies have so far reported conflicting results. Most studies were limited by lack of information on dosage and duration of use of the different classes of NSAIDs.

**Methods:**

We conducted a nested case-control study using data from Saskatchewan Prescription Drug Plan (SPDP) and Cancer Registry to examine the effects of dose and duration of use of five classes of NSAIDs on prostate cancer risk. Cases (N = 9,007) were men aged ≥40 years diagnosed with prostatic carcinoma between 1985 and 2000, and were matched to four controls on age and duration of SPDP membership. Detailed histories of exposure to prescription NSAIDs and other drugs were obtained from the SPDP.

**Results:**

Any use of propionates (e.g., ibuprofen, naproxen) was associated with a modest reduction in prostate cancer risk (Odds ratio = 0.90; 95%CI 0.84-0.95), whereas use of other NSAIDs was not. In particular, we did not observe the hypothesized inverse association with aspirin use (1.01; 0.95–1.07). There was no clear evidence of dose-response or duration-response relationships for any of the examined NSAID classes.

**Conclusions:**

Our findings suggest modest benefits of at least some NSAIDs in reducing prostate cancer risk.

## Introduction

It has been shown that non-steroidal anti-inflammatory drugs (NSAIDs) could prevent the development of colon cancer [1], and possibly other cancers [2], [3] including prostate cancer [4]. Proposed mechanisms for these effects, including induction of apoptosis [5] and inhibition of cellular proliferation and angiogenesis [6], occur at least partly through the inhibition of the cyclooxygenase (COX) enzymes involved in prostaglandin synthesis. Over-expression of COX-2 has been observed in prostate cancer cells [7], and higher levels of prostaglandins have been detected in malignant compared to benign prostate tissues [8]. In all 12 animal studies included in a recent review, NSAIDs exhibited inhibitory effects on prostate cancer development and progression to invasive disease [9].

Despite strong laboratory evidence, epidemiological studies of NSAID use and prostate cancer have so far produced conflicting results [4], [10], [11]. Although most studies reported inverse associations between aspirin use and prostate cancer occurrence, some found positive [12] or no associations [13], [14], [15], [16]. Studies that examined the effect of aspirin use on the occurrence of advanced prostate cancer were more consistent [12], [13], [14], [17], [18], [19]. Studies that examined the effects of non-aspirin (NA-NSAIDs) were inconsistent with cohort studies generally showing no association and case-control studies suggesting statistically significant inverse associations [4].

Most reviewed studies were limited by exposure and disease misclassification, by limited information on dose and duration of use and by the possibility of screening and other biases [4]. Also, there have been no studies that assessed the effects of individual classes of NSAIDs.

We assessed the effects of dose and duration of use of five chemical classes of NSAIDs on prostate cancer risk using a nested case-control analysis of a historical cohort that was assembled by means of record linkage of several large longitudinal databases of routinely collected health data from the Canadian province of Saskatchewan. To our knowledge, this is the largest study to specifically examine the hypothesis that NSAIDs may reduce the risk of prostate cancer, and the first study in the field to systematically examine the effects of five different classes of NSAIDs on prostate cancer risk, rather than just examine the effects of all NSAIDs or one NSAID.

## Methods

### Ethics statement

This study was approved by the Ethics Review Boards of McGill University and the University of Saskatchewan. Both boards deemed that obtaining consent from individual participants was not necessary or feasible because this study was based on the analysis of anonymous records obtained from administrative databases that include information on all residents of Saskatchewan.

### Data sources

Data were obtained by linkage of Saskatchewan Ministry of Health (SH) databases and the Saskatchewan Cancer Registry (SCR). SH provides publicly funded health insurance coverage, including coverage for prescription drugs and hospital and physician services, to most of the province’s one million residents. Eligibility for coverage is not based on age or income [20]. For administrative purposes, SH maintains several centralized electronic databases that can be linked using a unique health services number.

The Saskatchewan Prescription Drug Plan (SPDP), in operation since 1975, records all pharmacy claims for formulary drugs dispensed to Saskatchewan beneficiaries [20]. The accuracy of the recorded prescription information is high [21]. However, the SPDP lacks information on drugs given during hospitalization or bought over the counter (OTC).

All cancers occurring in the study cohort were identified using the population-based SCR, in operation since 1932. Because reporting of cancer cases is mandated by law, cancer registration is virtually complete in Saskatchewan [22]. Most (97%) cases are pathologically-confirmed, and fewer than 3% of registrations originate from death certificates [22]. For the cases, we also had access to detailed clinical information, including stage, Gleason score and results of PSA testing, which was obtained by abstracting clinical charts of all included prostate cancer cases as part of another research project. The methods of that project are documented in detail elsewhere [23].

Information on comorbidity and indication of NSAID use (e.g., diabetes, ischemic heart disease, arthritis, and prostatitis) and on utilization of health care services including urological procedures (Table 1) was obtained from SH hospital separation and physician services databases which, since 1971, recorded most services provided by Saskatchewan hospitals and physicians. The collected data include diagnostic and treatment information including a primary diagnosis, coded using the International Classification of Diseases, Ninth Revision (ICD-9), and service or procedure codes [20]. We used several previously validated algorithms [24] to identify cases of chronic diseases in our cohort (Table 1).

**Table 1:** Definitions of variables used in the analysis.

Variable	Definition
**NSAID classes** a	
Arylacetic acids	Diclofenac (100), Etodolac (400), Indomethacin (100), Sulindac (400), Tolmetin (700), Zomepirac (300)
Butylpyrazolidines	Phenylbutazone (300)
Fenamates	Floctafenine (1000), Mefenamic Acid (1000)
Oxicams	Meloxicam (15), Piroxicam (20)
Propionates	Fenoprofen (1200), Flurbiprofen (200), Ibuprofen (1200), Ketoprofen (150), Nabumetone (1000), Naproxen (500)
Coxibs	Celecoxib (200), Rofecoxib (20)
Aspirin (3000)	
**Screening**	
SCREENED	Binary variable with 1 indicating whether at any point prior to the index date a subject had a physician visit for BPH (ICD-9 code 600.*), prostatitis (601.*) or “other disorders of prostate” (602.*); or any point during the 11 years prior to the index date, a subject received at least one prescription for finasteride or an α-blocker or had prostatic ablation or resection, or testing of prostatic secretions. We assumed the men who received these services had at least a DRE.
**Medical conditions** b	
Diabetes	≥2 physician claims with ICD-9 = 250
Hypertension	≥2 physician claims with ICD-9 = 401, 405 OR ≥2 prescriptions for selective β-blockers; thiazides; CCBs-DH; or centrally acting anti-adrenergics
Rheumatoid arthritis	≥2 physician claims with ICD-9 = 714 OR ≥2 prescriptions for DMRDs or steroids
Osteoarthritis	≥3 physician claims with ICD-9 = 710–713; 715–739; No DMRD or steroids
Other inflammatory arthritis	≥3 physician claims with ICD-9 = 710–713; 715–739 AND ≥1 DMRD or steroids
Cardiac disease/stroke	≥3 physician claims with ICD-9 = 390–400;402–404;406–459
GI bleeding	≥1 physician claims with ICD-9 = 578
Prostatic hypertrophy	≥1 physician claims with ICD-9 = 600 OR ≥1 prescriptions for finasteride or α-blockers OR ≥1 TURP or ablation
Prostatitis	≥1 physician claims with ICD-9 = 601 OR ≥1 physician claims for MEPS.
**Others**	
Income status	Binary variable with 1 indicating ever having a prescription flagged for receiving income security benefits.
Vasectomy, TURP, Prostatic biopsy, MEPS	Information on these procedures was extracted from a list of all physician-provided urological services (services for which a physician claimed a fee-for-service code under section R of the Saskatchewan Ministry of Health’s “Payment Schedule for Insured Services Provided by a Physician”) since January 1, 1975.
Classes of medications	Prostatism agents, androgen antagonists, Lipid lowering agents, Angiotensin converting enzyme inhibitors, Angiotensin receptor blockers, α- and β-blockers, Antihypertensive calcium channel blockers, Centrally acting antihypertensives, Vasodilators, Diuretics, DMRDs, Systemic steroids, Anticoagulants, Cardiac glycosides, Proton pump inhibitors, H2 receptor antagonists, Other ulcer-healing agents. All drugs were classified according to the WHO ATC classification (see text).

a) For each NSAID, the WHO’s defined daily dose (DDD) used in the analysis is listed in parenthesis (in milligrams). The DDD is “the assumed average maintenance dose per day for a drug used for its main indication in adults”(WHO Collaborating Centre for Drug Statistics Methodology, 2002). Using DDDs, we effectively weighted the prescribed quantity of each NSAID by its anti-inflammatory potency.

b) Based on the most valid chronic disease identification algorithms (those algorithms with the highest Kappa and Youden’s index values) from a comprehensive review of the literature performed by Lix et al (20).

BPH: Benign prostate hypertrophy; CCBs-DH: Calcium channel blockers, dihydropyridine; DMRD: Disease-modifying anti-rheumatic drugs; DRE: Digital rectal examination; GI: Gastro-intestinal; MEPS: Microscopic examination of prostatic secretions; TURP: Transurethral resection of prostate.

### Historical cohort

The study cohort consisted of all men aged 40 years or older who were registered with SH during 1985–2000. Cohort members were followed from the latest of the study start date (January 1, 1985), their 40th birthday or the date of immigration to Saskatchewan until the study end date (December 31, 2000), or the date of diagnosis of prostate cancer, death or emigration, whichever occurred first. The population registry of SH, which tracks eligibility for health insurance coverage [20], was used to determine cohort members’ vital and migration status.

### Definition of cases and controls

To be eligible for inclusion in the nested case-control analysis, a participant must have been (1) free of cancer (except non-melanoma skin cancer) before the *index date*, defined as the date of diagnosis for a case or the date of diagnosis of the matching case for a control; and (2) a beneficiary of prescription drug coverage for a minimum of 5 years prior to his index date (to ensure that all participants had a reasonable opportunity to fill NSAID prescriptions before the index date). Registered Indians and other federal beneficiaries (9% of the population) were excluded because information about their drug use is not captured in the SPDP [20].

The cases group included all men (N = 9,007) in the study cohort who had a diagnosis of primary prostatic carcinoma (ICD-Oncology code C61; morphology codes: 8140/3, 8010/3, and 8000/3). Using incidence density sampling [25], we randomly selected up to four controls (N = 35,891) for each case from among eligible cohort members, matched on age (±1 year) and duration of SPDP membership.

### Measurement of prescription drug use

For each participant, detailed histories of exposure to dispensed NSAIDs and 18 other drug classes were obtained from the SPDP for the period between January 1, 1976, or the coverage initiation date, whichever was later, and the index date. The length of these histories was ≥10 years in 98.5% of participants (median 19, range 5–27). The WHO Anatomic Therapeutic Chemical (ATC) classification [26] was used to classify drugs, e.g., NSAIDs were defined as all drugs in the Saskatchewan drug formulary with ATC codes M01A* or N02BA*. NSAIDs were further classified into seven different chemical classes (Table 1). To facilitate comparisons with previous studies, NSAIDs were also classified, in separate analyses, into aspirin and non-aspirin NSAIDs (NA-NSAIDs).

Exposure to each class of NSAIDs was characterized in two ways: (1) as a binary (“any use”) variable indicating whether a participant ever filled a prescription of any drug in the index class at any time during his exposure history. (2) As an ordinal variable representing the quintiles of the average annual dose of the index class calculated by dividing the total dispensed quantity of the class by its overall duration of use (measured from the dispensing date of the first prescription that included a drug in that class). All drug use in the year immediately prior to the index date was excluded to avoid protopathic bias [27].

Because different drugs in the same class may have different pharmacologic potency, the total dispensed quantity for each drug was expressed as a proportion of the WHO’s defined daily dose (DDD) for that drug before summing up all these proportions as the total dispensed quantity of the class (see Table 1 for list of DDDs). The DDD is “the assumed average maintenance dose per day for a drug used for its main indication in adults” [26]. In most analyses, the average annual dose was categorized using the quintiles of the distribution, which were calculated after excluding observations with zero annual dose (“non-users”). Therefore, this variable had six levels: the five categories formed by the quintile cutoff points and a reference category formed by non-users.

We did not have information on the daily dose or duration of treatment as recommended by the prescribing clinician. To measure the duration of use, we relied on the fact that for most regular NSAID users, prescriptions were typically filled every 3 months. So for every participant, we divided the exposure history into 3-month periods beginning at the date of first prescription filled by that participant. We then counted the number of such periods that included at least 1 prescription. The duration of use variable (in years) was then computed as the sum of these 3-month periods, and further categorized into 7 categories: 0, 0.25, 0.5, 0.75–1.5, 1.75–3.0, 3.25–6.0 and ≥6.25 years, with cutoff points corresponding to the 50^th^, 75^th^, 90^th^, 95^th^, 99^th^ centiles of the duration of aspirin use variable.

### Statistical analysis

We used conditional logistic regression (CLR) to model the effects of NSAID use on prostate cancer risk while accounting for matching and other confounding variables. The final models were adjusted for screening predictors and, when appropriate, for use of other classes of NSAIDs.

We lacked information on PSA testing among the controls; so instead we adjusted for three variables believed to be associated with heightened screening [28]: ever having seen a urologist in the 1–11 years prior to the index date (i.e. excluding the year immediately prior to the index date); volume of family physician visits in the 5 years prior to the index date; and a composite binary variable (SCREENED) which took the value of 1 if a participant was diagnosed with a prostatic condition other than prostate cancer or received a diagnostic or therapeutic intervention for such a condition (see Table 1 for details). Consistent with strong correlation with screening status, these variables were associated with increased detection of early prostate cancers and reduced detection of advanced prostate cancers.

We also performed a forward step-wise empirical search for confounders. A variable was considered a confounder if its inclusion in adjusted models resulted in a>2% change in OR estimates of any of the study’s main exposures. Using this criterion, none of the variables considered, including a large number of medications (e.g., finasteride, statins) and indications of NSAID use (see Table 1 for a list of these variables), was deemed an empirical confounder, and were therefore excluded from the final models.

We used incremental odds ratios (iORs) to assess for monotonic linear dose-response relationships between the quintiles of the average annual dose and prostate cancer risk. Unlike conventional ORs which contrast the risk at each exposure level with the same reference category, iORs are derived using models that contrast the effect at each level with that at the previous level [29]. Therefore, iORs consistently (at all levels) above (or below) 1.0 suggest a monotonic increasing (or decreasing) dose-response relationship. The confidence intervals around these iORs provide a measure of the statistical significance of these trends.

Given the long exposure histories in this cohort, the NSAID users group will naturally include participants with highly variable exposure histories. To reduce the effect of this heterogeneity, and to assess for the presence of an “induction period” for NSAID effects (the time interval between an exposure exerting its causal effects and disease initiation or prevention [30]), analyses were repeated after dividing the exposure history into six successive periods: the first spanned the 12-month period prior to the index date. The other periods spanned 5 years each and were as follows: 1.1–6, 6.1–11, 11.1–16, 16.1–21, 21.1–26 years. A separate exposure index was computed for each period by limiting exposure measurements to prescriptions dispensed during that period [31]. As before, CLR models were used to estimates ORs associated with drug use in each period with mutual adjustment for exposure in other periods as well as adjustment for screening predictors.

## Results

Most (80%) cases were older than 65 (median age  = 73), and were mostly (83%) diagnosed during the 1990s, following the widespread use of PSA screening. At diagnosis, 12% of cases had locally-invasive disease (Whitmore-Jewett stage C) and another 15% had metastases (stage D). Gleason score was greater than 7 in 14% of cases.

Overall, 82.2% of cases and 79.5% of controls have received at least one NSAID prescription (Table 2). Propionates, arylacetic acids and aspirin were the most commonly prescribed NSAIDs. Ignoring matching, there were no significant differences between cases and controls in the median number of filled prescriptions for any of the examined classes (Table 2).

**Table 2:** Ever-use of NSAIDs and descriptive statistics of total number of prescriptions among ever-users by case-control status and drug category.

Drug category^a^	No (%) of ever-users	No. of prescriptions among ever-users					
		Mean	SD	Q1	Median	Q3	Max
**Aspirin**							
Controls	17469(48.7)	6.5	13.0	1.0	2.0	5.0	238.0
Cases	4653(51.7)	6.6	13.6	1.0	2.0	5.0	172.0
**Coxibs**							
Controls	100(0.3)	3.4	3.0	1.0	2.0	5.0	14.0
Cases	27(0.3)	3.1	2.2	1.0	3.0	4.0	9.0
**Arylacetic acids**							
Controls	16779(46.7)	7.3	14.2	1.0	3.0	6.0	283.0
Cases	4442(49.3)	7.1	13.2	1.0	3.0	6.0	141.0
**Butylpyrazolidines**							
Controls	6102(17.0)	2.6	4.6	1.0	1.0	2.0	122.0
Cases	1657(18.4)	2.6	4.1	1.0	1.0	2.0	81.0
**Fenamates**							
Controls	833(2.3)	2.5	7.5	1.0	1.0	2.0	95.0
Cases	234(2.6)	1.9	3.3	1.0	1.0	2.0	43.0
**Oxicams**							
Controls	5976(16.7)	4.9	9.1	1.0	2.0	4.0	164.0
Cases	1620(18.0)	5.0	9.2	1.0	2.0	4.0	100.0
**Propionates**							
Controls	18667(52.0)	6.4	12.3	1.0	2.0	6.0	208.0
Cases	4881(54.2)	6.1	11.1	1.0	2.0	6.0	151.0
**NA-NSAIDs**							
Controls	25542(71.2)	11.3	19.8	2.0	4.0	11.0	330.0
Cases	6683(74.2)	11.1	18.2	2.0	4.0	11.0	216.0
**NSAIDs**							
Controls	28516(79.5)	14.2	23.5	2.0	6.0	15.0	372.0
Cases	7406(82.2)	14.2	22.5	2.0	6.0	15.0	295.0

a) See Table 1 for a list of drugs in each category.

SD: standard deviation; Q1 and Q3: First and third quartiles.

In models accounting for matching but not adjusting for any other confounders (Table 3, left panel), ever filling an NSAID prescription was associated with a small increase in risk (odds ratio [OR] = 1.21; 95%CI 1.13–1.28). Similar results were observed for the different classes of NSAIDs, including aspirin (1.13; 1.08–1.18) and propionates (1.10; 1.05–1.15).

**Table 3:** Effect of ever filling a prescription of an NSAID class on the risk of developing prostate cancer.

Variable	Unadjusted OR^a^ (95%CI)	P-value	Adjusted OR^b^ (95%CI)	P-value
Aspirin	1.13 (1.08–1.18)	<0.001	1.01 (0.95–1.07)	0.816
Arylacetic acids	1.11 (1.06–1.17)	<0.001	0.94 (0.88–1.00)	0.043
Butylpyrazolidines	1.10 (1.04–1.17)	0.002	0.99 (0.92–1.07)	0.776
Oxicams	1.10 (1.03–1.17)	0.002	0.96 (0.89–1.04)	0.368
Propionates	1.10 (1.05–1.15)	<0.001	0.89 (0.84–0.95)	<0.001
Coxibs	1.09 (0.70–1.69)	0.712	Excluded^c^	
Fenamates	1.12 (0.97–1.30)	0.119	Excluded^c^	
NA-NSAIDs	1.17 (1.11–1.24)	<0.001	0.88 (0.82–0.94)^d^	<0.001
NSAIDs	1.21 (1.13–1.28)	<0.001	0.87 (0.80–0.94)	<0.001

a) ORs from unadjusted conditional logistic regression models for comparison.

b) Adjusted for ever visited a urologist 1–11 years prior, SCREENED and volume of family physician visits in the 5 years prior to the index date and, when appropriate, for use of other NSAID classes.

c) Fenamates and Coxibs were excluded from this model because of small numbers.

d) From an adjusted model that included terms for NA-NSAIDs and aspirin in addition to screening predictors as above.

Note: Effect estimates throughout the paper have been rounded to two decimal digits. This is not meant to imply that our results are accurate to two decimal digits (most certainly they are not). However, rounding to one single digit would have made it difficult to spot any trends in the data.

Following adjustment for screening and aspirin use (Table 3, right panel), any use of NA-NSAIDs was inversely associated with prostate cancer risk (0.88; 0.82–0.94). In a model with mutual adjustment for 5 NSAID classes, propionates (0.89; 0.84–0.95) and arylacetic acids (0.94; 0.88–1.00) were inversely associated with disease risk whereas any use of aspirin was not (OR = 1.01 [95%0.95–1.07]).

A similar pattern was observed when exposure was represented as the quintiles of the average annual dose. Table 4 shows the results from two separate models that included mutual adjustment for quintiles of the average annual dose of five NSAID classes. In one model, dose quintiles were entered as an ordinal variable (a linear term). In the second, each level of the ordinal dose variable was represented in the model with a binary indicator variable. The OR associated with the linear term of aspirin annual dose was 0.99 (0.97–1.01). Aspirin use was not statistically significantly associated with prostate cancer at any dose level. On the other hand, propionate use was inversely associated with prostate cancer risk; linear term = 0.97 (0.96–0.99). Inverse associations were seen at all levels above 1.1 DDDs/year, but there was no clear evidence of a monotonic dose-effect relationship.

**Table 4:** Effect of average annual NSAID dose (in DDDs/year; categorized using the quintiles of the distribution) on the risk of developing prostate cancer.

Variable^a^	OR (95%CI)^b^	P-value	iOR^c^ (95%CI)	P-value
**Aspirin**				
Linear term	0.99 (0.98–1.01)	0.380		
Quintiles				
Never-users	1.0 (reference)			
0.1–0.1	1.04 (0.94–1.16)	0.422	1.04 (0.94–1.16)	0.422
0.2–0.6	1.08 (0.97–1.19)	0.146	1.03 (0.91–1.18)	0.617
0.7–1.7	0.97 (0.87–1.07)	0.547	0.90 (0.79–1.02)	0.102
1.8–4.9	1.02 (0.92–1.13)	0.734	1.05 (0.92–1.19)	0.448
≥5.0	0.93 (0.84–1.03)	0.158	0.91 (0.80–1.03)	0.147
**Arylacetic acids**				
Linear term	0.99 (0.97–1.01)	0.292		
Quintiles				
Never-users	1.0 (reference)			
0.1–1.0	0.94 (0.85–1.05)	0.290	0.94 (0.85–1.05)	0.290
1.1–2.2	0.91 (0.82–1.01)	0.073	0.96 (0.84–1.10)	0.573
2.3–4.6	0.95 (0.86–1.05)	0.321	1.04 (0.92–1.19)	0.526
4.7–12.3	0.94 (0.85–1.05)	0.284	0.99 (0.87–1.13)	0.927
≥12.4	0.97 (0.87–1.09)	0.615	1.03 (0.90–1.17)	0.648
**Butylpyrazolidines**				
Linear term	1.00 (0.98–1.02)	0.932		
Quintiles				
Never-users	1.0 (reference)			
0.1–0.5	0.99 (0.85–1.16)	0.929	0.99 (0.85–1.16)	0.929
0.6–0.8	1.03 (0.88–1.20)	0.751	1.03 (0.83–1.28)	0.767
0.9–1.5	0.98 (0.84–1.14)	0.782	0.95 (0.77–1.18)	0.667
1.6–3.0	0.86 (0.74–1.01)	0.070	0.88 (0.71–1.10)	0.263
≥3.1	1.11 (0.95–1.30)	0.197	1.28 (1.04–1.59)	0.023
**Oxicams**				
Linear term	0.99 (0.96–1.01)	0.254		
Quintiles				
Never-users	1.0 (reference)			
0.1–1.4	1.06 (0.90–1.23)	0.491	1.06 (0.90–1.23)	0.491
1.5–2.4	0.97 (0.83–1.14)	0.699	0.92 (0.74–1.14)	0.439
2.5–4.4	0.99 (0.85–1.16)	0.929	1.02 (0.82–1.27)	0.825
4.5–10.6	0.88 (0.75–1.03)	0.120	0.89 (0.72–1.10)	0.280
≥10.7	0.95 (0.81–1.11)	0.516	1.07 (0.87–1.33)	0.504
**Propionates**				
Linear term	0.97 (0.96–0.99)	0.004		
Quintiles				
Never-users	1.0 (reference)			
0.1–1.1	0.92 (0.83–1.01)	0.092	0.92 (0.83–1.01)	0.092
1.2–2.5	0.88 (0.80–0.98)	0.017	0.97 (0.85–1.10)	0.588
2.6–5.1	0.91 (0.82–1.01)	0.072	1.03 (0.91–1.17)	0.641
5.2–13.2	0.88 (0.79–0.97)	0.012	0.96 (0.85–1.09)	0.543
≥13.3	0.89 (0.80–1.00)	0.042	1.02 (0.90–1.16)	0.757

a) For each class, results from two separate models are reported. In one model, the dose quintiles were entered as an ordinal variable (a linear term). In the second model, each level (quintile) of the ordinal dose variable was represented in the model with a binary indicator variable. In the analyses shown in the left panel, the reference group is men who did not fill any prescriptions of the index class (never-users).

b) Adjusted for ever having visited a urologist 1–11 years prior, SCREENED and volume of family physician visits in the 5 years prior to the index date, and for all NSAID classes listed in the table.

c) iOR: Incremental OR. The ORs in the right panel are incremental ORs from models that contrast the effect at each level with that at the previous level. iORs consistently (at all levels) above (or below) 1.0 suggest a monotonic increasing (or decreasing) dose-response relationship.

Similar results (data not shown) were obtained when the average annual dose variables were categorized using “fixed” cutoff points that were all multiples of 10 DDDs/year, (i.e., 2.5, 5, 10, 20 and 40; 10 DDDs/year of NSAID use is equivalent to 1 year use of a once daily dose of 81 milligrams of aspirin). Specifically, for each NSAID class, the annual average dose was categorized into 0 (never-use), 0.1–2.4, 2.5–4.9, 5.0–9.9, 10.0–19.9, 20.0–39.9 and 40.0–79.9 DDDs/year. In these analyses, inverse associations at all levels were observed for propionates. However, there was no clear monotonic dose-effect relationship demonstrated in any of these analyses.

As shown in Table 5, duration of use of aspirin was not associated with prostate cancer risk (linear term OR = 0.99 [0.97–1.02]). Although all levels of the propionate duration of use variable were inversely associated with disease risk, the associations were generally not statistically significant, and there was no clear trend of stronger associations with longer duration of use.

**Table 5:** Effect of duration of NSAID prescriptions (in years) on the risk of developing prostate cancer.

Variable^a^	OR (95%CI)^b^	P-value	iOR^c^ (95%CI)	P-value
**Aspirin**				
Linear term	0.99 (0.97–1.02)	0.594		
Categories				
Never-users	1.0 (reference)			
0.25	1.03 (0.96–1.12)	0.381	1.03 (0.96–1.12)	0.381
0.5	1.14 (1.03–1.26)	0.014	1.10 (0.98–1.23)	0.097
0.75–1.5	0.96 (0.87–1.06)	0.400	0.84 (0.74–0.95)	0.007
1.75–3.0	0.87 (0.75–1.01)	0.059	0.91 (0.77–1.06)	0.233
3.25–6.0	0.91 (0.77–1.07)	0.262	1.05 (0.85–1.29)	0.668
>6.0	1.26 (1.00–1.57)	0.047	1.38 (1.06–1.81)	0.018
**Arylacetic acids**				
Linear term	1.00 (0.97–1.02)	0.656		
Categories				
Never-users	1.0 (reference)			
0.25	0.92 (0.85–1.00)	0.057	0.92 (0.85–1.00)	0.057
0.5	0.97 (0.87–1.08)	0.577	1.05 (0.94–1.18)	0.402
0.75–1.5	0.96 (0.88–1.06)	0.428	0.99 (0.88–1.12)	0.897
1.75–3.0	1.02 (0.89–1.17)	0.801	1.06 (0.91–1.22)	0.452
3.25–6.0	0.91 (0.77–1.08)	0.295	0.89 (0.73–1.10)	0.281
>6.0	1.03 (0.82–1.30)	0.789	1.13 (0.86–1.48)	0.368
**Butylpyrazolidines**				
Linear term	0.99 (0.95–1.03)	0.660		
Categories				
Never-users	1.0 (reference)			
0.25	0.99 (0.90–1.09)	0.888	0.99 (0.90–1.09)	0.888
0.5	0.92 (0.78–1.08)	0.315	0.93 (0.77–1.11)	0.403
0.75–1.5	0.94 (0.79–1.13)	0.517	1.03 (0.81–1.30)	0.837
1.75–3.0	1.41 (0.99–2.02)	0.058	1.50 (1.01–2.23)	0.044
3.25–6.0	0.85 (0.46–1.56)	0.601	0.60 (0.30–1.21)	0.156
>6.0	0.29 (0.06–1.37)	0.118	0.34 (0.06–1.81)	0.204
**Oxicams**				
Linear term	0.97 (0.94–1.01)	0.099		
Categories				
Never-users	1.0 (reference)			
0.25	1.02 (0.92–1.13)	0.686	1.02 (0.92–1.13)	0.686
0.5	0.92 (0.78–1.08)	0.283	0.90 (0.75–1.08)	0.241
0.75–1.5	0.85 (0.72–0.99)	0.035	0.92 (0.74–1.14)	0.467
1.75–3.0	0.92 (0.72–1.18)	0.525	1.09 (0.82–1.45)	0.547
3.25–6.0	1.04 (0.73–1.48)	0.817	1.13 (0.74–1.73)	0.575
>6.0	0.76 (0.36–1.60)	0.468	0.73 (0.32–1.65)	0.448
**Propionates**				
Linear term	0.98 (0.96-1.00)	0.029		
Categories				
Never-users	1.0 (reference)			
0.25	0.91 (0.84–0.99)	0.024	0.91 (0.84–0.99)	0.024
0.5	0.96 (0.87–1.07)	0.483	1.06 (0.95–1.18)	0.301
0.75–1.5	0.88 (0.80–0.96)	0.005	0.91 (0.81–1.02)	0.099
1.75–3.0	0.92 (0.80–1.06)	0.235	1.05 (0.91–1.21)	0.509
3.25–6.0	0.93 (0.78–1.11)	0.444	1.01 (0.83–1.24)	0.891
>6.0	0.87 (0.68–1.13)	0.298	0.94 (0.70–1.26)	0.662

a) For each class, results from two separate models are reported. In one model, the duration of use categories were entered as an ordinal variable (a linear term). In the second model, each level of the ordinal duration of use variable was represented in the model with a binary indicator variable. In the analyses shown in the left panel, the reference group is men who did not fill any prescriptions of the index class (never-users).

b) Adjusted for ever having visited a urologist 1–11 years prior, SCREENED and volume of family physician visits in the 5 years prior to the index date, and for all NSAID classes listed in the table.

c) iOR: Incremental OR.

Table 6 shows results of models that included period-specific binary terms for ever-use of each of five classes of NSAIDs. The aim of these analyses was to identify the exposure window (period) that is most likely associated with possible biological effects of NSAID use. The strongest inverse association for aspirin was seen for the period 1.1-6 years before the index date, but there was no discernable pattern to the period-specific ORs, and none of them was statistically significant. For propionates, the strongest inverse association was observed during the 11.1–16 years period, OR = 0.85 (95%CI 0.76–0.94). Strong positive associations were observed for several NSAIDs during the one-year period immediately before the index date, likely due to protopathic bias as NSAIDs are widely used to manage pain, which could be a symptom of undetected cancer. Similar pattern of results was observed when the linear (ordinal) term of the average annual dose (as defined in the dose-effect analysis) was substituted for the binary ever-use term (data not shown).

**Table 6:** Effect of NSAID ever-use in each exposure period (in years before the index date) on the risk of developing total prostate cancer by NSAID category.

Variable	OR (95%CI)^a^	P-value
**Aspirin**		
≤1	1.00 (0.87–1.16)	0.954
1.1–6	0.93 (0.83–1.03)	0.157
6.1–11	0.96 (0.87–1.06)	0.409
11.1–16	1.02 (0.91–1.15)	0.677
16.1–21	1.10 (0.96–1.27)	0.161
21.1–26	1.00 (0.79–1.26)	0.990
**Arylacetic acids**		
≤1	1.20 (1.06–1.37)	0.005
1.1–6	0.99 (0.91–1.08)	0.796
6.1–11	0.99 (0.90–1.09)	0.813
11.1–16	0.94 (0.84–1.05)	0.277
16.1–21	0.96 (0.83–1.12)	0.642
21.1–26	0.77 (0.57–1.05)	0.098
**Butylpyrazolidines**		
≤1	1.35 (0.45–4.05)	0.589
1.1–6	1.25 (0.89–1.76)	0.200
6.1–11	1.13 (0.91–1.41)	0.262
11.1–16	0.79 (0.65–0.97)	0.022
16.1–21	0.99 (0.80–1.23)	0.931
21.1–26	1.05 (0.78–1.42)	0.734
**Oxicams**		
≤1	0.90 (0.61–1.31)	0.567
1.1–6	0.96 (0.81–1.15)	0.683
6.1–11	1.01 (0.87–1.18)	0.886
11.1–16	0.96 (0.81–1.14)	0.674
16.1–21	0.84 (0.63–1.12)	0.234
**Propionates**		
≤1	1.11 (0.96–1.30)	0.164
1.1–6	0.93 (0.85–1.01)	0.098
6.1–11	0.96 (0.88–1.05)	0.426
11.1–16	0.85 (0.76–0.94)	0.001
16.1–21	1.05 (0.92–1.20)	0.439
21.1–26	0.87 (0.68–1.12)	0.291

a) Adjusted for ever visited a urologist 1–11 years prior, SCREENED and volume of family physician visits in the 5 years prior to the index date, and to all terms listed in the table.

## Discussion

We found that propionate use was consistently inversely related to prostate cancer risk whereas aspirin use was not. The strongest association was observed with propionate use taking place 11–16 years before diagnosis.

Although the bulk of the literature is suggestive of protective effects for aspirin use [4], our results are consistent with those from four large population-based cohort studies [13], [14], [15], [16] in showing no benefits. Also, ours is not the only analysis where a small aspirin-propionate difference was noted. Harris et al. reviewed the evidence for the effect of NSAID use on 10 cancer sites, and concluded that compared to aspirin and other NSAIDs, ibuprofen (a propionate) has a stronger anti-cancer effect [32]. Very few studies have specifically examined the effects of propionate use on prostate cancer [13], [33], and their findings were generally consistent with ours.

The lack of inverse association with aspirin use may have been due to disease misclassification. Under-ascertainment of cases could occur if some cancer cases were not captured by the SCR or if occult prostate cancer, common among older men [34], was under-detected. The errors are likely non-differential with respect to NSAID use, and could bias our ORs towards the null [35].

However, differential misclassification due to screening is likely a more significant concern. NSAID users are more likely to be screened, probably because of more frequent contacts with health care providers [28], [36]. One major limitation of SH databases is the lack of information on PSA testing. As a workaround, we used several predictors of screening to adjust our models for the effect of screening [35]. These adjustments resulted in the expected (downward) correction in the crude estimates. However, it is possible that these predictors may have been misclassified with respect to the participants’ true screening status. However, disease misclassification does not explain the observed inverse association with propionate use.

Errors in the measurement of NSAID use are another concern. We assumed that the amount of NSAIDs dispensed is a good approximation of actual consumption, which is likely true for chronic users with repeated refills. We also lacked information on non-prescription use, e.g., medications bought over the counter. However, except for aspirin and ibuprofen, the non-prescribed amounts are probably very small compared to the amounts of prescribed medications [37].

Several lines of evidence suggest that misclassification due to lack of information on OTC aspirin and ibuprofen use did not lead to significant bias. There was no change in risk estimates for ibuprofen use when we limited the analysis to cases diagnosed before August 1989, the year when ibuprofen became available without prescription in Saskatchewan. Similarly, ORs for aspirin use did not change appreciably when we stratified the analysis by markers of OTC aspirin use such as history of ischemic heart disease and diabetes. Also, any such bias is likely non-differential. So it could have biased the ORs towards the null, but that would not explain the inverse associations observed for propionates (including ibuprofen) use.

In addition, we used a Monte Carlo sensitivity analysis to assess the potential effects of exposure measurement errors on study estimates. Using multiple imputation and simulation methods, levels of aspirin and ibuprofen use were adjusted to reflect both random and systematic sources of underascertainment of their use [38]. Regardless of NSAID type, exposure index or the assumed error rate, we saw no significant differences from the empirical estimates (data available on request). For propionates, all levels of the average annual dose quintile variable remained inversely related to prostate cancer risk, especially with higher simulated use rates. For both aspirin and propionates, the linear trends were smoother than those observed empirically.

The possibility of confounding should also be considered. We found no evidence that any of a large number of medications and indications and contraindications of NSAID use (Table 1) was a significant confounder. We could not adjust for ethnicity. However, the generation of men included in this study was predominately Caucasian (most Aboriginal men were excluded), thus any confounding effect is likely small [39]. We have no reason to believe that family history of prostate cancer could be a confounder. We lacked information on putative lifestyle risk factors. In previous studies, adjustment for these factors did not appreciably change the crude estimates [13], [19], [40]. This is not surprising given the lack of known significant exogenous risk factors for prostate cancer [41]. Although we cannot rule out the possibility of bias due to residual confounding, our sensitivity analyses suggest that even a strong confounder (one associated with a 5-fold increase or decrease in prostate cancer risk) will not fully explain the observed differences between aspirin and propionate use.

The aspirin-propionate differences may also stem from differences in patterns of use of these medications. Among older men, aspirin is prescribed in low doses primarily for cardio-protection whereas propionates are used in full strength doses as analgesic and anti-inflammatory medications. However, our dose-response analysis suggests that propionate use was inversely related to prostate cancer risk at all dose levels whereas aspirin was not.

Finally, the observed differences between aspirin and propionates could be due to genuine heterogeneity in effect reflecting differences in their pharmacokinetics or biological effects. For instance, compared to other NSAIDs, aspirin undergoes an extensive first pass hepatic metabolism following oral administration [19], which may translate into lower availability at the tissue level inside the prostate gland. Aspirin is a potent inhibitor of COX-1 whereas the propionates are potent inhibitors of both COX-1 and COX-2 [42]. This is important because the bulk of the evidence from laboratory studies is consistent with a more important role for COX-2 in prostatic carcinogenesis [7], [43]. Lastly, it has been suggested that some NSAIDs may have anti-tumour effects independent of COX blockade [44]. For instance, R-flurbiprofen and exisulind, NSAIDs that are not active against COX, have significant anti-neoplastic properties [45]. Also, some anti-tumour effects of NSAIDs are not reversed by the addition of prostaglandins, or seem to occur at tissue concentrations lower than those required for COX inhibition [44]. It is plausible that NSAIDs differ in their ability to induce these COX-independent effects, which may explain some of the differences observed in this study.

In conclusion, we found that use of propionates was associated with a small reduction in prostate cancer risk. There was no clear evidence of dose-response or duration-response relationships with any of the examined NSAID classes. Further studies are needed to confirm the observed associations, and to address important unanswered questions about the specific NSAIDs with the largest benefits, and the optimal dose and duration of use required for maximum benefits [46]. Compared to other novel chemopreventive agents, the toxicity profiles of the classic NSAIDs are generally well understood [1]. However, any potential benefits of NSAID use would have to be carefully weighed against the risks associated with their regular use [47].
